# Supplementary material for: Cover Crops Modulate the Response of Arbuscular Mycorrhizal Fungi to Water Supply: A Field Study in Corn
Source: Plants (Basel). 2023 Feb 23;12(5):1015. doi: 10.3390/plants12051015 (PMC10005079; doi:10.3390/plants12051015)
Supplement: Supplementary file 1 [file plants-12-01015-s001.zip › plants-2201928-supplementary.pdf]

SUPPLEMENTARY MATERIAL

Cover crops modulate the response of arbuscular mycorrhizal fungi to water supply: a field study in corn

Micaela Tosi[1], Cameron M. Ogilvie[2], Federico N. Spagnoletti[3,4], Sarah Fournier[1], Ralph C. Martin[2], Kari E. Dunfield[1*]

[1] School of Environmental Sciences, University of Guelph, 50 Stone Rd. E, Guelph, ON N1G 2W1, Canada

[2] Department of Plant Agriculture, University of Guelph, 50 Stone Rd. E, Guelph, ON N1G 2W1, Canada

[3] CONICET-Consejo Nacional de Investigaciones Científicas / Instituto de Investigaciones en Biociencias Agrícolas y Ambientales, Avda. San Martín 4453, C1417DSE, Buenos Aires, Argentina

[4] Cátedra de Microbiología, Facultad de Agronomía, Universidad de Buenos Aires, Buenos Aires, Argentina

** Corresponding authors: dunfield@uoguelph.ca*

SUPPLEMENTARY FIGURES

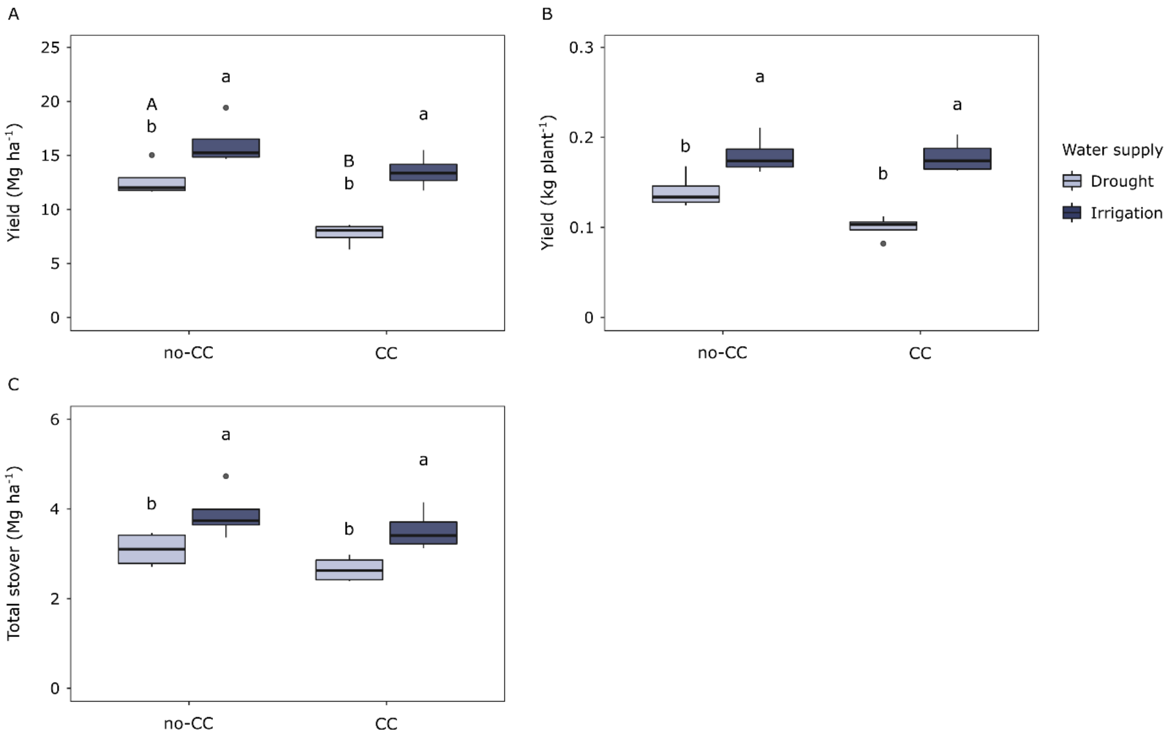

Figure S1. Corn yield per hectare (A) and per plant (B), and corn stover biomass (C) in response to cover crops (no-CC: no cover crop, CC: 4-species cover crop) and water supply (drought, irrigation). Different lowercase letters show significant differences between water supply treatments for each CC treatment, and different uppercase letters in A show CC effects for drought treatment (Tukey test, alpha=0.05).

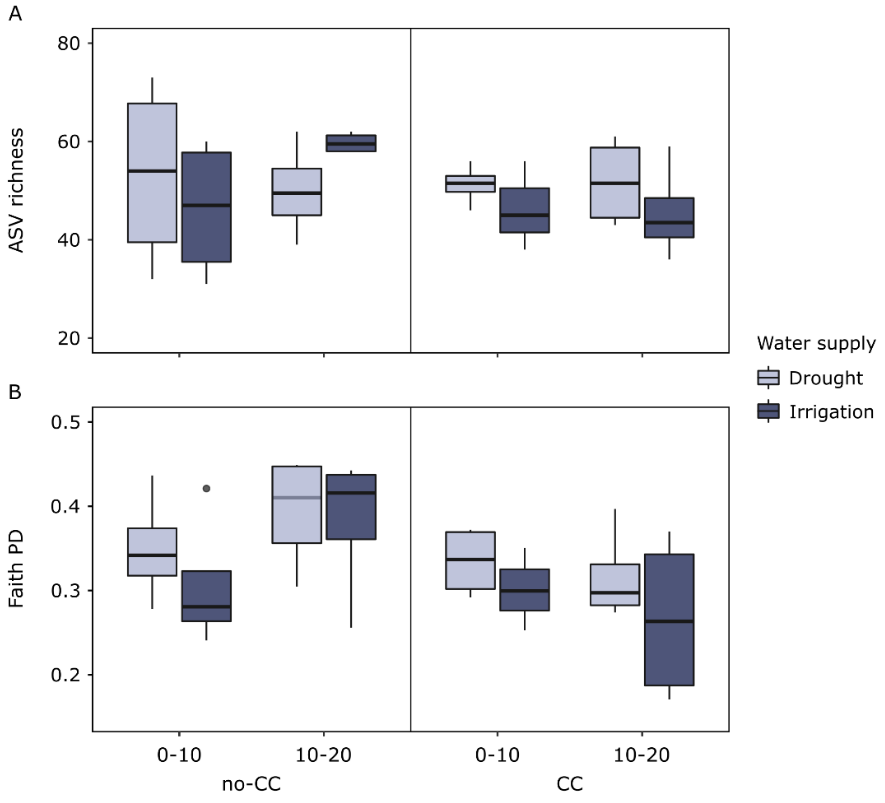

Figure S2. Arbuscular mycorrhizal fungi (AMF) ASV richness (A) and phylogenetic richness as Faith PD (B) in response to cover crops (no-CC: no cover crop, CC: 4-species cover crop) and water supply (drought, irrigation). Data are shown for two different soil depths (0-10 and 10-20 cm). No significant water supply or CC effects were detected (ANOVA and Tukey test, alpha=0.05).

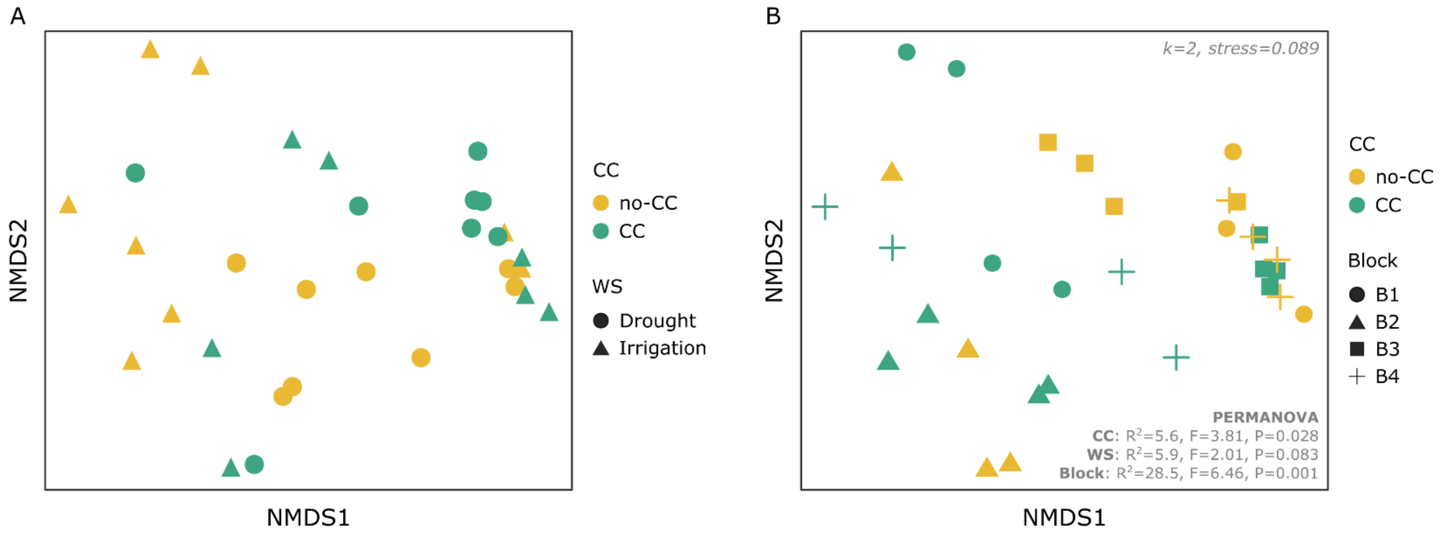

Figure S3. Non-metric multidimensional scaling (NMDS) showing changes in soil arbuscular mycorrhizal fungi (AMF) community composition (weighted UniFrac) in response to cover crop (CC) treatments. The same analysis is shown twice: in A, shapes indicate water supply (WS) treatments, whereas in B, they indicate field block ids. NMDS dimensions (k) and stress, as well as CC, WS and block effects according to PERMANOVA (R^2: % variance explained, F value and P value) are shown on the left figure. Full PERMANOVA results table can be found in Table S2.

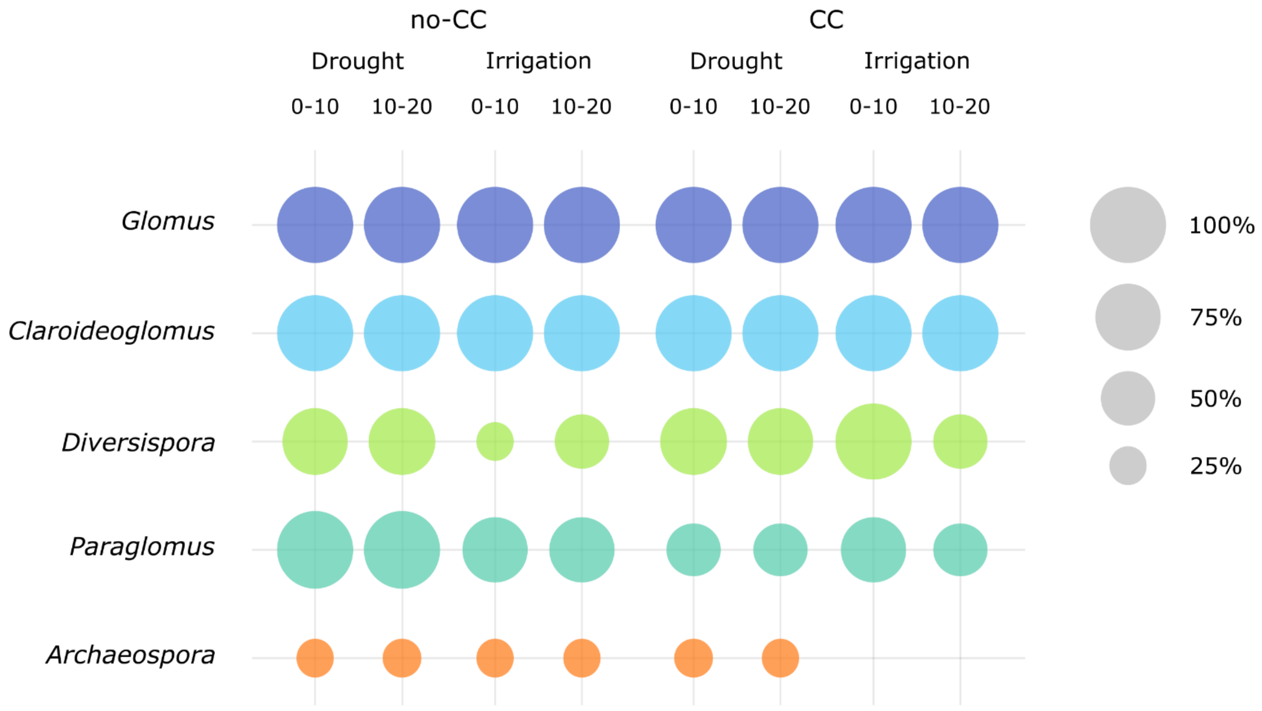

Figure S4. Prevalence of different soil AMF genera in the different cover crop treatments (no-CC: no cover crop, CC: 4-species cover crop), water supply treatments (drought, irrigation) and soil depths (0-10 and 10-20 cm). Prevalence is presented as percentage out of the 4 plots or field replicates sampled for each treatment.

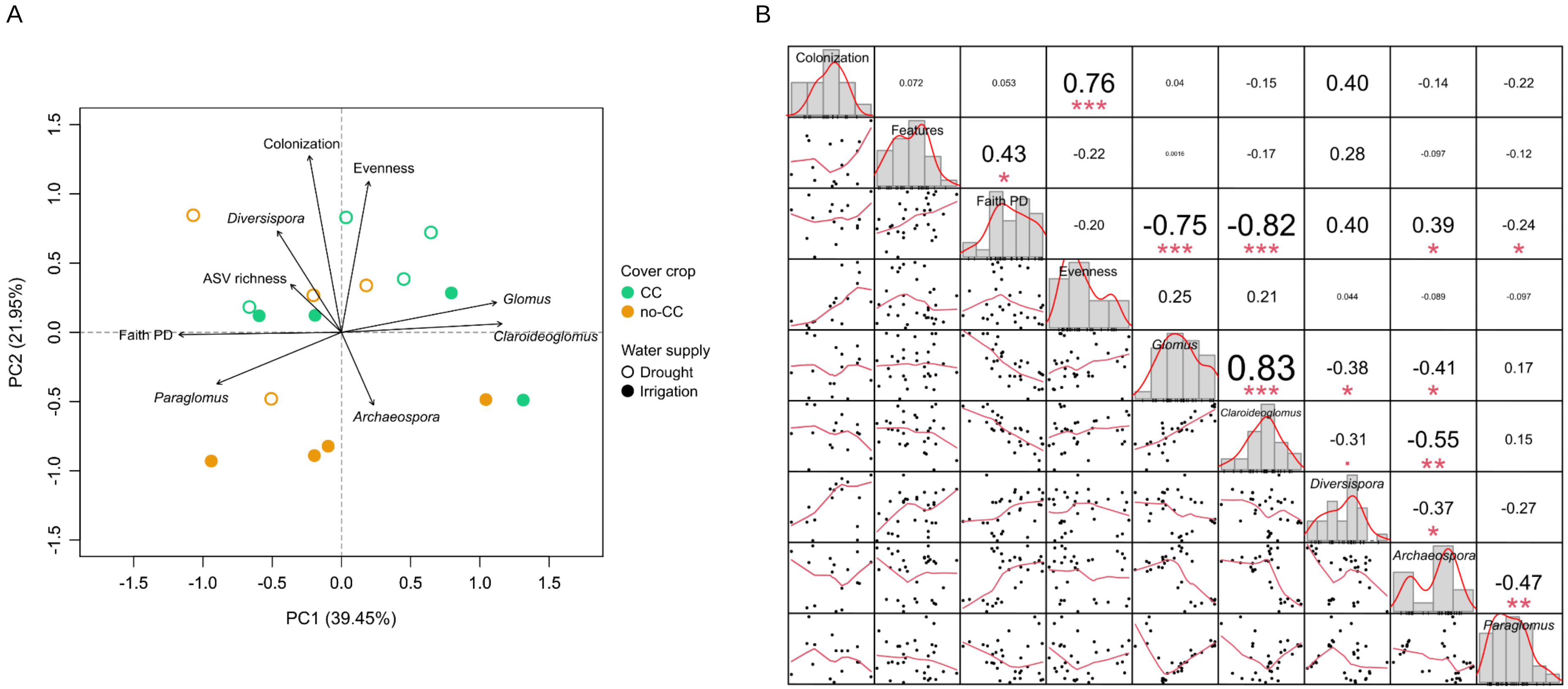

Figure S5. Principal component analysis (PCA) (A) and Spearman correlation coefficients (B) showing the relationship between the main arbuscular mycorrhizal fungi (AMF) variables measured in this study: AMF colonization of corn roots, ASV richness and Faith PD of soil AMF communities, and relative abundance of AMF taxa in soil (transformed to centered log ratio). Sites are coloured by cover crop (no-CC: no cover crop, CC: 4-species cover crop) and symbol types represent water supply (drought, irrigation) treatments. Correlation analysis results can be found in Table S3.

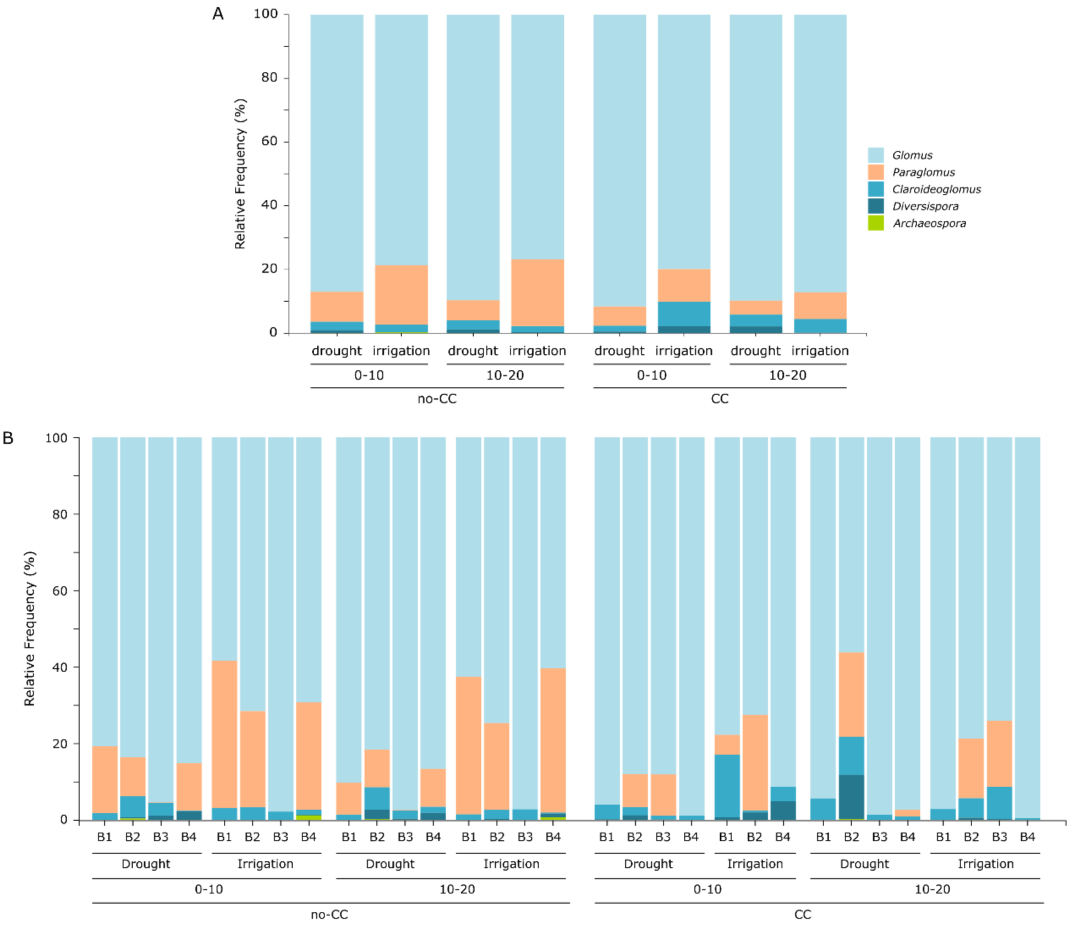

Figure S6. Taxa barplots showing relative frequency (%) of arbuscular mycorrhizal fungi (AMF) genera in soils under different cover crop (no-CC: no cover crop, CC: 4-species cover crop) and water supply (drought, irrigation) treatments, and from two different soil depths (0-10 and 10-20 cm). Plot A shows data after pooling all blocks or field replicates in a treatment, whereas plot B shows each individual field replicate.

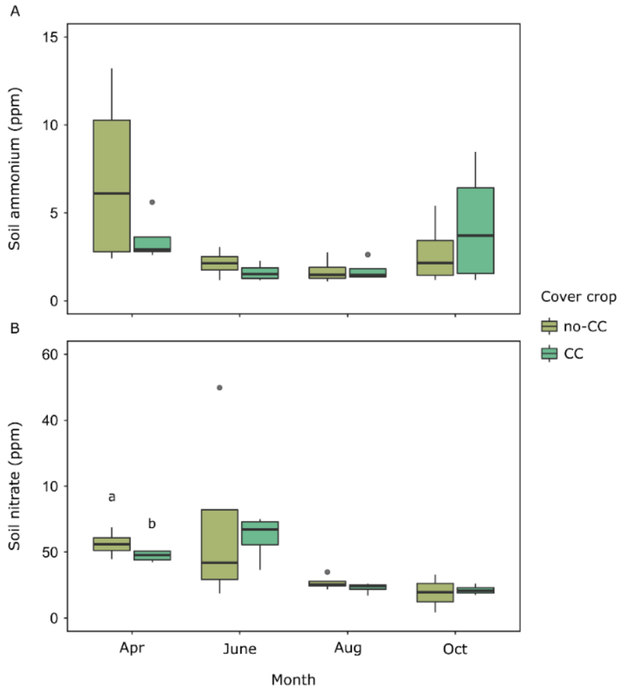

Figure S7. Ammonium (A) and nitrate (B) content in soils under different cover crop (no-CC: no cover crop, CC: 4-species cover crop) treatments. In B, different letters who significant differences between CC treatments in each sampling month (Tukey test, alpha=0.05).

SUPPLEMENTARY TABLES

Table S1. ANOVA results testing effect of cover crop (CC) and water supply (WS) on three variables of arbuscular mycorrhizal fungi (AMF) colonization of corn roots (all in %): overall colonization (hyphae), arbuscules and vesicles.

		numDF	denDF	F-value	p-value
Colonization	(Intercept)	1	16	357.47	<.0001
	CC	1	3	1.20	0.3528
	WS	1	6	19.16	**0.0047**
	CC:WS	1	6	1.30	0.2981
Arbuscules	(Intercept)	1	16	38.79	<.0001
	CC	1	3	1.70	0.2839
	WS	1	6	10.21	**0.0187**
	CC:WS	1	6	3.11	0.1283
Vesicles	(Intercept)	1	16	419.40	<.0001
	CC	1	3	4.02	0.1387
	WS	1	6	19.36	**0.0046**
	CC:Water	1	6	2.84	0.1431

Table S2. PERMANOVA results showing effects of cover crops (CC) and water supply (WS) on soil AMF phylogenetic community composition. Results are shown for a quantitative and a qualitative phylogenetic distance metric (weighted and unweighted, respectively), both for the overall analyses and different subsets of data.

		weighted UniFrac				unweighted UniFrac			
		CC	WS	CC*water	block	CC	WS	CC*water	block
Overall test	R^2	5.61	5.90	4.13	28.49	6.98	2.45	6.74	31.46
	F	3.81	2.01	1.41	6.46	5.03	0.89	2.44	7.60
	P	**0.028**	0.083	0.222	**0.001**	**0.001**	0.542	**0.013**	**0.001**
WS test (no-CC)	R^2		15.95		63.63		5.57		56.91
	F		8.60		11.43		1.63		5.56
	P		**0.002**		**0.001**		0.156		**0.001**
WS test (CC)	R^2		6.28		69.14		5.61		62.35
	F		2.56		9.38		1.75		6.48
	P		0.085		**0.001**		0.139		**0.001**
CC test (drought)	R^2	7.30			44.26	11.71			44.81
	F	1.65			3.35	2.96			3.78
	P	0.170			**0.011**	**0.028**			**0.001**
CC test (irrigated)	R^2	13.85			30.48	12.79			43.90
	F	2.48			1.83	2.95			3.38
	P	0.111			0.159	**0.039**			**0.005**

R^2: variance explained, in %
Because depth and its interactions with other variables were never significant, final analyses were carried out ignoring this variable.

Table S3. ANOVA and Tukey test P-values from soil moisture as volumetric water content (VWC) analyses showing water supply (WS) and cover crop (CC) effects. Analyses were carried out separately for each month (July, August and September) and each soil depth (5 and 20 cm). Table shows P-value from ANOVA (overall) and from Tukey test within each level of the second treatment (e.g., water supply effects within each CC level). The arrows indicate if the effect was positive (↑) or negative (↓).

		**Interaction**	**WS effects**			**CC effects**		
			**Overall**	**no-CC**	**CC**	**Overall**	**Drought**	**Irrigation**
July	5 cm	*n.s.*	↑ 0.004	↑ 0.005	↑ *0.073*	*n.s.*	*n.s.*	*n.s.*
	20 cm	*n.s.*	↑ 0.021	↑ 0.041	*n.s.*	*n.s.*	*n.s.*	*n.s.*
Aug	5 cm	0.041	↑ 0.001	↑ 0.001	↑ 0.019	*n.s.*	*n.s.*	*n.s.*
	20 cm	*n.s.*	↑ *0.094*	*n.s.*	*n.s.*	*n.s.*	*n.s.*	*n.s.*
Sep	5 cm	0.087	↑ 0.008	↑ 0.007	*n.s.*	*n.s.*	*n.s.*	↓ *0.098*
	20 cm	*n.s.*	↑ 0.012	↑ 0.032	↑ 0.039	*n.s.*	*n.s.*	*n.s.*

The July category also includes June 28-30.
